# Evaluation and identification of powdery mildew-resistant genes in 137 wheat relatives

**DOI:** 10.3389/fgene.2024.1342239

**Published:** 2024-01-24

**Authors:** Jiaojiao Wang, Hongxing Xu, Yanmin Qie, Ran Han, Xiaohui Sun, Ya Zhao, Bei Xiao, Zejun Qian, Xiaomei Huang, Ruishan Liu, Jiadong Zhang, Cheng Liu, Yuli Jin, Pengtao Ma

**Affiliations:** ^1^ Yantai Key Laboratory of Characteristic Agricultural Bioresource Conservation & Germplasm Innovative Utilization, College of Life Sciences, Yantai University, Yantai, China; ^2^ School of Life Sciences, Henan University, Kaifeng, Henan, China; ^3^ Institute of Cereal and Oil Crops, Hebei Academy of Agricultural and Forestry Sciences, Hebei Key Laboratory of Crop Genetics and Breeding, Shijiazhuang, China; ^4^ Crop Research Institute, Shandong Academy of Agricultural Sciences, Jinan, China; ^5^ Institute of Grain and Oil Crops, Yantai Academy of Agricultural Science, Yantai, China

**Keywords:** powdery mildew, markers, resistance identification, haplotype analysis, wheat relatives

## Abstract

Powdery mildew is one of the most severe diseases affecting wheat yield and quality and is caused by *Blumeria graminis* f. sp. *tritici* (*Bgt*). Host resistance is the preferred strategy to prevent this disease. However, the narrow genetic basis of common wheat has increased the demand for diversified germplasm resources against powdery mildew. Wheat relatives, especially the secondary gene pool of common wheat, are important gene donors in the genetic improvement of common wheat because of its abundant genetic variation and close kinship with wheat. In this study, a series of 137 wheat relatives, including 53 *Triticum monococcum* L. (2n = 2x = 14, AA), 6 *T. urartu Thumanjan ex* Gandilyan (2n = 2x = 14, AA), 9 *T. timopheevii* Zhuk. (2n = 4x = 28, AAGG), 66 *T. aestivum* subsp*. spelta* (2n = 6x = 42, AABBDD), and 3 *Aegilops speltoides* (2n = 2x = 14, SS) were systematically evaluated for their powdery mildew resistance and composition of *Pm* genes. Out of 137 (60.58%) accessions, 83 were resistant to *Bgt* isolate E09 at the seedling stage, and 116 of 137 (84.67%) wheat relatives were resistant to the mixture of *Bgt* isolates at the adult stage. This indicates that these accessions show a high level of resistance to powdery mildew. Some 31 markers for 23 known *Pm* genes were used to test these 137 accessions, and, in the results, only *Pm2*, *Pm4*, *Pm6*, *Pm58*, and *Pm68* were detected. Among them, three *Pm4* alleles (*Pm4a*, *Pm4b*, and *Pm4f*) were identified in 4 *T*. subsp*. spelta* accessions. q-RT PCR further confirmed that *Pm4* alleles played a role in disease resistance in these four accessions. The phylogenetic tree showed that the kinship of *Pm4* was close to *Pm24* and *Sr62*. This study not only provides reference information and valuable germplasm resources for breeding new wheat varieties with disease resistance but also lays a foundation for enriching the genetic basis of wheat resistance to powdery mildew.

## Introduction

Wheat (*Triticum aestivum* L.) is one of the foremost staple crops in the world, contributing significantly to global food security (Igrejas and Branlard, 2020). However, its productivity is consistently threatened by myriad biotic and abiotic factors, among which wheat powdery mildew is prominent. Powdery mildew, caused by *Blumeria graminis* f. sp. *tritici* (*Bgt*), primarily affects wheat leaves by severely impairing photosynthesis, nutrient uptake, and overall yield, leading to diminished grain quality and quantity. In severe cases, yield losses can reach up to 40% (Savary et al., 2019). Traditionally, the management of wheat powdery mildew has relied on chemical pesticides, which present challenges such as environmental pollution and high costs. Therefore, more environmentally friendly, economical, and sustainable approaches are needed to control wheat powdery mildew. The development of wheat cultivars with inherent resistance to powdery mildew has emerged as a pivotal solution to combat this issue.

Since the first cataloged wheat powdery mildew resistance (*Pm*) gene *Pm1* was identified in 1952, 69 formally cataloged *Pm* genes have been successively reported in the primary, secondary, and tertiary gene banks of wheat (McIntosh et al., 2020; He et al., 2021; Li et al., 2023). Among them, the 13 *Pm* genes *Pm1*-*5*, *Pm8*, *Pm12*, *Pm17*, *Pm21*, *Pm24*, *Pm41*, *Pm60*, and *Pm69*, and two multiple resistance loci *Pm38*/*Yr18*/*Lr34*/*Sr57/Ltn1/Sb1* and *Pm46*/*Yr46*/*Lr67*/*Sr55/Ltn3* have been cloned (Krattinger et al., 2009; Brunner et al., 2010; Hurni et al., 2013; Moore et al., 2015; Sánchez-Martín et al., 2016; Singh et al., 2018; Xing et al., 2018; Zou et al., 2018; Li et al., 2020; Lu et al., 2020; Xie et al., 2020; Hewitt et al., 2021; Sánchez-Martín et al., 2021; Li et al., 2023; Zhu et al., 2023). Although so many *Pm* genes have been reported, most of them cannot be directly used for production because of linkage drag or other bad agronomic traits, such as *Pm55* derived from *Dasypyrum villosum* L. and *Pm56* derived from rye (*Secale cereale* L.) (Zhang et al., 2016; Hao et al., 2018). Therefore, a long-term goal for fighting this disease is to mine more novel genes against powdery mildew and apply them in wheat breeding projects.

As an allohexaploid species, common wheat has three subgenomes (2n = 6x = AABBDD) derived from three wheat relatives over the long process of domestication. The wheat relatives constitute one or two subgenomes of wheat which constitute the secondary gene banks for wheat improvement. In modern wheat breeding, due to long-term artificial selection, the primary gene pool of common wheat has been fully developed and utilized, making it difficult to breed new breakthrough wheat cultivars. In comparison with primary gene banks, secondary gene banks may have larger genetic variation. Compared to tertiary gene banks, genes derived from secondary gene banks can be more fully recombined into the wheat genome, avoiding the problem of poor genetic compensation which often occurs in breeding utilization of tertiary gene sources in the form of translocations. Therefore, the unique characteristics of secondary gene sources make it easier to achieve a balance between comprehensive agronomic traits and disease resistance in production. For example, *Pm4* from *T. monococcum* L. (2n = 2x = 14, AA), *Pm6* from *T. timopheevii* Zhuk (2n = 4x = 28, AAGG), and *Pm50* from *T. dicoccum* L. (2n = 4x = 28, AABB) were all introduced into common wheat, significantly improving wheat resistance to powdery mildew (Mohler et al., 2013; Wan et al., 2020; Sanchez-Martin et al., 2021). The *Aegilops* and *Triticum* genera are quite similar in genetic relationship and are easy to cross with common wheat. They are also widely used in the genetic improvement of wheat. For example, *Pm13* on chromosome 3S^l^ of *Ae. longissima* has been applied in wheat disease resistance breeding (Zhang et al., 2014). *Ae. speltoides* (2n = 2x = 14, SS) is a donor of tetraploid and hexaploid. *Pm12*, *Pm32*, and *Pm53* derived from *Ae*. *speltoides*, which have also been introduced into wheat’s genetic background (Hsam et al., 2003; Petersen et al., 2015; Zhu et al., 2023).

Accurate identification of these resistance genes in specific genotypes is a key step in rapidly and effectively applying them in wheat breeding programs. Over recent decades, traditional breeding methods have developed some resistant wheat varieties, but they demand extensive time and effort. However, recent progress in molecular biology and molecular marker technology has provided novel tools and strategies in breeding for wheat disease resistance. Molecular marker detection is a technique based on wheat genomic DNA that enables the precise and efficient identification and selection of wheat plants that carry resistance genes. Since a growing number of resistance genes have been identified and cloned, and functional markers, which have been developed based on polymorphic SNPs/Indels within their full-length sequences, could directly detect genotype variations accurately in practical application (Liu et al., 2012). For resistance genes that have not been cloned, such as *Pm6*, *Pm58,* and *Pm68,* closely linked markers are the preferred method of detecting them, (Ji et al., 2008; He et al., 2021; Xue et al., 2022). Previous research has identified the resistance phenotypes and gene compositions in various germplasm resources. For instance, Gebrewahid et al. (2020) identified the leaf rust resistance and *Lr* genes in 50 bread wheat cultivars derived from Ethiopia by molecular marker analysis.

In our lab, 137 wheat relatives, including 53 *T. monococcum* L. (2n = 2x = 14, AA), 6 *T. urartu Thumanjan ex* Gandilyan (2n = 2x = 14, AA), 9 *T. timopheevii* Zhuk. (2n = 4x = 28, AAGG), 66 *T. aestivum* subsp*. spelta* (2n = 6x = 42, AABBDD), and 3 *Ae. speltoides* (2n = 2x = 14, SS) were provided by the International Maize and Wheat Improvement Center (CIMMYT). To systematically evaluate the powdery mildew resistance and explore elite *Pm* genes/alleles in these germplasms, we 1) investigated their powdery mildew resistance at the seedling and adult stages, 2) identified the composition of *Pm* genes by molecular marker detection, 3) analyzed the haplotype of the cloned *Pm* genes in these accessions, and 4) explored the relationship between *Pm* genes and disease resistance.

## Materials and methods

### Plant materials and pathogens

A total of 137 wheat relatives, including 53 *T. monococcum* L. (2n = 2x = 14, AA), 6 *T. urartu Thumanjan ex* Gandilyan (2n = 2x = 14, AA), 9 *T. timopheevii* Zhuk (2n = 4x = 28, AAGG), 66 *T. aestivum* subsp*. spelta* (2n = 6x = 42, AABBDD), and 3 *Ae. speltoides* (2n = 2x = 14, SS) were provided by CIMMYT and used to test their reaction patterns to the *Bgt* isolate E09 (Supplementary Table S1). The wheat cultivar Tainong 18 has been identified as being susceptible to all tested *Bgt* isolates, so it was used as the susceptible check in phenotype identification. Ten extra single spore-derived *Bgt* isolates—F01, F06, F19, F21, F26, F28, E17, E18, E21, and E23-1—with different avirulent/virulent patterns were used for testing against the reaction patterns of E09-resistant accessions. These isolates were preserved on the susceptible Tainong 18 seedlings which were placed in independent glass tubes. To avoid cross infection, these tubes were covered with three layers of gauze.

### Resistance identification to powdery mildew at the seedling stage

The 137 wheat relatives were first evaluated for their resistance to the *Bgt* isolate E09, a moderate virulent isolate mainly in the wheat production regions of North China. The resistant genotypes were further tested for their resistance to ten extra *Bgt* isolates. The reaction patterns of the tested accessions were determined in the greenhouse of Yantai University, Yantai, China. Five to eight seeds of each accession were sown in a 72-cell (4.0 × 4.0 × 4.2 cm) rectangular tray (54 × 28 × 4.2 cm). In the sowing design, Tainong 18, as the susceptible check, was planted randomly with three cells in each tray. These trays were placed into separate growth chambers with a daily cycle of 22°C/14 h/light and 18°C/10 h/darkness and were infected with different virulent *Bgt* isolates. Two weeks later, when the seedlings had grown to the one-leaf and one-heart stage, they were inoculated with fresh conidiospores propagated on Tainong 18 seedlings and cultured in conditions of 18°C/24 h/darkness with 100% humidity and then cultivated with a daily cycle of 22°C/14 h/light and 18°C/10 h/darkness. About 2 weeks later, when the spores had fully spread on the first leaf of Tainong 18, infection types (ITs) on each seedling were estimated based on a 0–4 scale. The evaluation standard was described as per Si et al. (1992). Among them, ITs 0, 1, and 2 were regarded as resistant genotypes, and ITs 3 and 4 as susceptible genotypes. All experimental procedures were repeated three times to ensure the accuracy and reliability of the data.

### Resistance identification to powdery mildew at the adult stage

The 137 wheat relatives were sown in the experiment field at Yantai University (Yantai, China, 121.39′E, 37.52′N) to evaluate their resistance to powdery mildew at the adult stage in 2021–2023. In mid-October of each year, ten seeds per accession were bunch-planted, with a 0.30-m space between bunches. Tainong 18, as the susceptible check, was sown around the plots to promote the spread of spores. In early April of the next year, when the temperature had successively risen above 10°C, Tainong 18 was inoculated with a mixture of *Bgt* isolates (including E09, F01, F06, F19, F21, F26, F28, E17, E18, E21, and E23-1). They were then irrigated to maintain humidity to promote the onset of powdery mildew. In early June, when Tainong 18 was highly susceptible, ITs of the tested genotypes were assessed and recorded twice at an interval of 7–10 days on the 0–9 scale, in which ITs 0–2 were regarded as highly resistant, ITs 3 and 4 as moderately resistant, ITs 5 and IT 6 as moderately susceptible, and ITs 7–9 as highly susceptible (Saari and Prescott, 1975).

### Molecular marker detection

Genomic DNA was isolated from the young seedlings of the tested genotypes using the protocol in Sharp et al. (1988) with minor modification. Specific primers of 23 reported *Pm* genes (*Pm1*-*5*, *Pm6*, *Pm8*, *Pm12*, *Pm21*, *Pm24*, *Pm35*, *Pm37*, *Pm41*, *Pm42*, *Pm45*, *Pm47*, *Pm52*, *Pm58*, *Pm59*, *Pm60*, *Pm61*, *Pm68*, and *Pm69*) were used to test all 137 wheat accessions in this study (Supplementary Table S2). PCR amplification was performed according to Jin et al. (2023). PCR products of most primers were detected in 8% non-denaturing polyacrylamide gels with 19:1, 29:1, or 39:1 ratios of acrylamide and bis-acrylamide, and were silver stained (Santos et al., 1993). The rest of the PCR products were separated by agarose gel with a concentration of 1.5% with nucleic acid dyestuff ultra GelRed (Vazyme Biotech Co., Ltd. China), and were then observed using the Gel Documentation System (Gel Doc XR+, BIO-RAD, Hercules, CA, United States) (Gebrewahid et al., 2020). See Supplementary Table S2 for the marker information and corresponding positive control of tested *Pm* genes.

### Homologous cloning and sequence analysis

Total RNA was isolated from the young leaf tissues using TRIzol reagent (Invitrogen, United States) following product instructions. Residual DNA was then removed, and the corresponding cDNA was synthesized using the FastKing gDNA Dispelling RT SuperMix kit (Tiangen) thus: a 10-µL reaction mixture containing 50 ng–2 µg RNA, 2 µL 5× gDNA buffer and ddH_2_O to 10 μL, 42°C for 3 min, then adding 2 µL 10× King RT buffer, 1 µL FastKing RT Enzyme Mix, 2 µL FQ-RT Primer Mix and ddH_2_O to 10 μL, 42°C for 15 min, 95°C for 3 min. The *Pm4* alleles were first cloned using primers GH398/GH399, GH400/GH401, GH398/GH407, and GH407/GH400 by nested PCR (Supplementary Table S2) and were then sequenced by Sanger sequencing (Sangon Biotech) and compared to the reported *Pm4a-4e* (GenBank, *Pm4b_V1*: MT783929, *Pm4b_V2*: MT783930) alleles with Seqman software (DNASTAR, Madison, American). For the new haplotypes, the DNA sequences were translated to amino acids with Editseq software (DNASTAR, Madison, American) for further analysis. The cDNA sequences of cloned disease-resistant genes were taken from the National Centre for Biotechnology Information (NCBI) database and were used for analysis by constructing the phylogenetic tree using the neighbor-joining method with the Poisson model in the MEGA7 software (Kumar et al., 2016).

### qRT-PCR analysis

The qRT-PCR procedure was performed using ChamQ universal SYBR qPCR Master Mix (Vazyme, China) on the Roche LightCycler^®^ 480 II real-time PCR system (Roche, Switzerland) with the specific primers *Pm4-qPCR* and *TaActin*. The expression pattern of each gene was calculated as a fold change using the comparative CT method (Livak and Schmittgen, 2001). Three biological replicates and three technical replications were performed to ensure data reliability. The *TaActin* gene was used as the standardized internal control.

## Results

### Phenotype identification against *Bgt* isolates at the seedling stage

**FIGURE 1:**
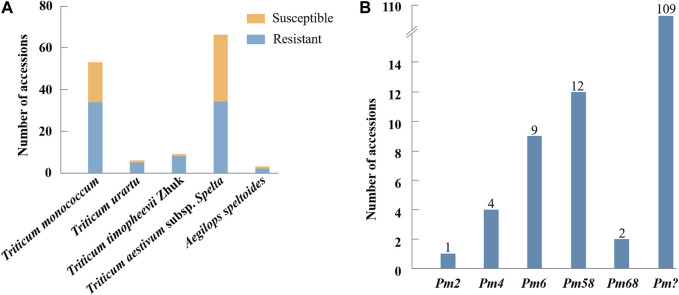
Resistance assessment of 137 wheat relatives to *Blumeria graminis* f. sp. *tritici* isolate E09 at the seedling stage **(A)**. Molecular marker detection of 137 wheat relatives **(B)**. “?” indicates untested *Pm* genes in the present study.

**FIGURE 2:**
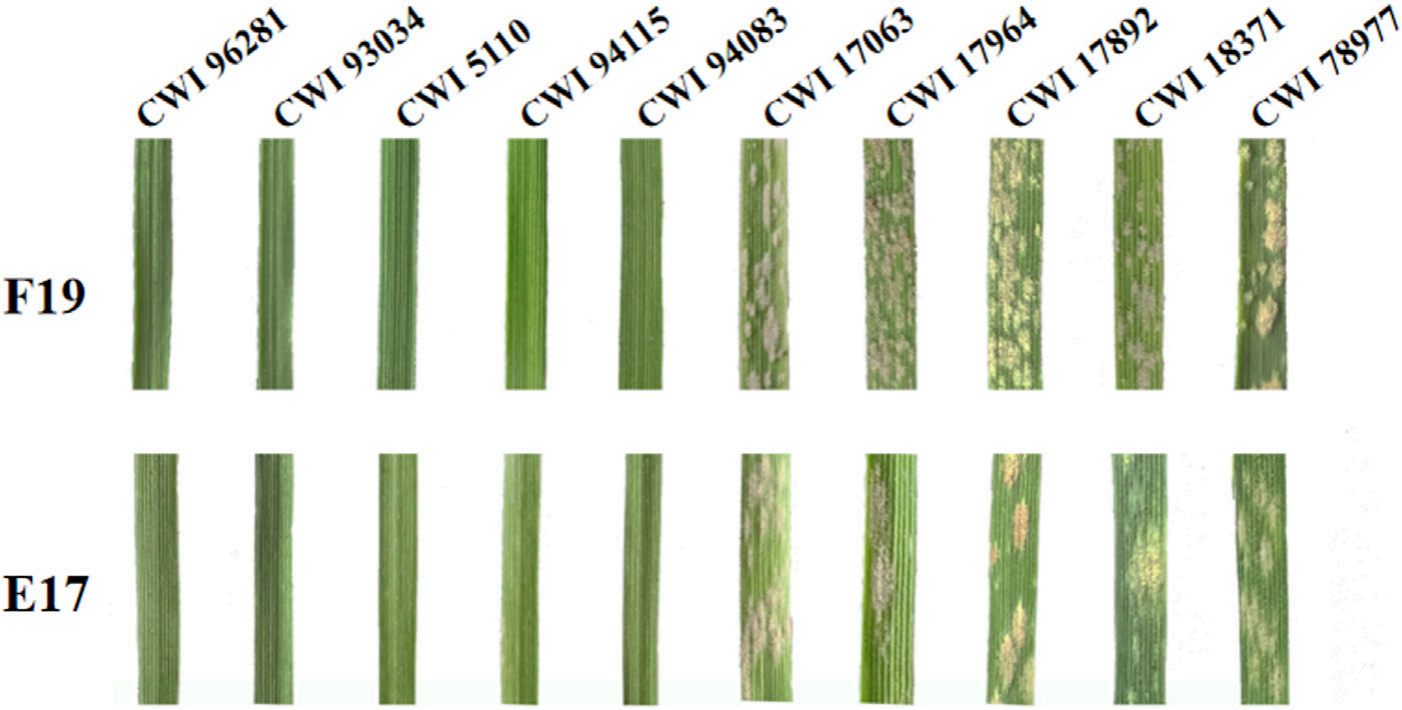
Reaction patterns of *T. monococcum* L. (CWI 96281, CWI 93034, CWI 5110, CWI 94115, and CWI 94083) and *T. aestivum* subsp. *spelta* (CWI 17063, CWI 17964, CWI 17892, CWI 18371, and CWI 78977) with powdery mildew *B. graminis* f. sp. *tritici* (*Bgt*) isolates F19 and E17.

When inoculated with the *Bgt* isolate E09, 83 of 137 (60.6%) wheat relatives, including 34 out of 53 *T. monococcum* L., 5 of 6 *T. urartu Thumanjan ex* Gandilyan, 8 of 9 *T. timopheevii* Zhuk., 34 of 66 *T. aestivum* subsp*. spelta*, and 2 of 3 *Ae. speltoides* were resistant with ITs 0–2, whereas the remaining 54 (39.4%) were susceptible with ITs 3–4 (Figure 1A, Supplementary Table S1). The proportion of the resistant genotypes was high, and these accessions were expected to be valuable gene donors for wheat improvement against powdery mildew. Notably, among 83 resistant genotypes, 70 showed immunity with IT 0, suggesting their significant resistance against powdery mildew. Then, those 83 E09-resistant genotypes were tested on the resistance spectrum with ten extra *Bgt* isolates. The results showed that they have different reaction patterns to these ten *Bgt* isolates (Figure 2; Table 1). A total of 32 accessions showed resistance to the ten *Bgt* isolates tested, such as CWI 18477, CWI 96263, CWI 96237, CWI 96281, and CWI 96275; four were resistant to nine of the ten tested *Bgt* isolates; 70 showed resistance to tested *Bgt* isolates which were diversified from one to eight. However, 31 accessions were susceptible to all these *Bgt* isolates (Table 1). This suggests that these wheat relatives have abundant genetic diversity in responding to powdery mildew.

**TABLE 1:** Reaction patterns of 83 E09-resistant genotypes to 10 *Blumeria graminis* f. sp. *tritici*.

No.	Plant ID	*B. graminis* f. sp. *tritici* (*Bgt*) isolates									
		F01	F06	F19	F21	F26	F28	E17	E18	E21	E23-1
1	CWI 83497	0	0	0	0	2	0	0	0	0	0
2	CWI 96237	0	0	0	0	0	0	0	0	0	0
3	CWI 96263	0	0	0	0	0	0	0	0	0	0
4	CWI 96272	3	0	3	3	3	0	0	2	4	0
5	CWI 96275	0	1	0	0	0	0	1	0	1	0
6	CWI 96277	3	1	3	3	1	0	0	0	3	0
7	CWI 96279	0	0	0	0	0	0	0	0	0	0
8	CWI 96281	0	0	0	0	0	0	0	0	0	0
9	CWI 96353	3	3	0	0	0	1	3	1	0	3
10	CWI 17058	4	3	4	0	4	3	3	4	3	4
11	CWI 17154	4	4	4	0	3	0	0	1	1	3
12	CWI 19498	0	0	0	0	0	0	0	0	0	0
13	CWI 19535	0	1	0	0	0	0	1	0	0	0
14	CWI 2352	0	0	0	0	0	0	0	0	0	0
15	CWI 38331	0	0	0	0	1	0	0	0	0	0
16	CWI 5103	1	0	1	0	0	0	0	0	0	0
17	CWI 6265	0	0	0	0	0	0	0	0	0	0
18	CWI 17260	3	3	2	4	4	2	4	3	0	0
19	CWI 18643	2	1	1	0	2	3	1	1	2	1
20	CWI 18754	1	1	0	0	0	2	0	0	0	0
21	CWI 18758	1	0	1	0	0	0	0	0	0	0
22	CWI 4385	3	3	1	2	0	0	4	2	0	2
23	CWI 4416	2	2	0	2	0	0	0	0	0	0
24	CWI 5110	0	0	0	0	0	0	0	0	0	2
25	CWI 93034	0	0	0	0	0	0	0	0	0	0
26	CWI 94083	1	0	0	0	1	0	0	1	0	2
27	CWI 94105	0	0	1	1	1	1	1	0	1	1
28	CWI 94107	0	0	0	0	0	0	0	0	0	0
29	CWI 94109	1	0	1	1	1	2	0	1	2	1
31	CWI 94145	0	0	0	0	0	1	1	0	0	0
32	CWI 94149	1	1	1	1	0	0	1	1	1	0
33	CWI 94157	0	1	0	0	0	0	1	0	0	0
34	CWI 17195	0	0	0	0	0	0	0	0	0	1
35	CWI 52947	0	0	0	0	0	0	0	0	0	0
36	CWI 19082	0	2	0	0	2	1	3	2	2	0
37	CWI 19093	2	0	0	0	3	0	0	2	0	0
38	CWI 19047	1	1	0	0	2	0	0	0	0	0
39	CWI 19058	0	0	0	0	0	0	0	0	0	0
40	CWI 80427	0	0	0	1	0	0	0	0	0	0
41	CWI 17007	3 + 0	3 + 0	1 + 0	4 + 0	3 + 0	2 + 0	3 + 0	3 + 0	1 + 0	3 + 0
42	CWI 17224	3	2	2	2	3	3	3	3	1	3
43	CWI 17259	2	3	0	3	1	1	0	3	0	3
44	CWI 17281	3	0	3	3	3	0	4	3	0	0
45	CWI 18175	3	3	3	3	1	3	4	3	3	4
46	CWI 18564	1	3	1	4	2	3	4	2	3	3
47	CWI 18594	2	1	1	1	3	2	1	2	2	1
48	CWI 17063	4	4	4	4	4	4	4	4	4	4
49	CWI 17247	4	4	4	4	4	4	4	4	4	4
50	CWI 17677	4	3	4	4	4	4	4	4	4	4
51	CWI 17750	4	4	4	4	4	4	4	4	4	4
52	CWI 17879	4	4	4	4	4	4	4	4	4	3
53	CWI 17892	4	4	4	3	4	4	4	3	4	4
54	CWI 17954	4	4	4	4	4	4	4	4	3	4
55	CWI 17959	4	4	3	4	4	4	4	4	4	4
56	CWI 17964	4	4	4	4	4	4	4	3	4	4
57	CWI 18065	4	4	4	4	4	4	4	4	4	4
58	CWI 18080	4	4	4	4	4	4	4	4	4	4
59	CWI 18143	4	4	4	4	4	4	4	4	4	4
60	CWI 18165	4	4	4	4	4	4	4	4	4	4
61	CWI 18166	4	4	4	4	3	3	4	4	4	4
62	CWI 18353	4	4	3	3	4	4	4	4	4	4
63	CWI 18371	4	4	4	4	4	4	4	4	4	4
64	CWI 18434	3	3	3	4	4	4	4	4	3	3
65	CWI 19131	4	4	4	4	3	4	4	4	4	4
66	CWI 44154	3	4	3	3	4	4	4	4	4	4
67	CWI 44355	3	4	4	4	4	3	4	3	3	4
68	CWI 44396	4	4	4	4	4	3	4	3	3	4
69	CWI 44403	4	4	4	4	4	4	4	4	3	4
70	CWI 78972	4	4	4	4	4	4	4	4	3	4
71	CWI 78977	4	3	4	4	4	4	4	4	4	4
72	CWI 78979	4	4	4	4	4	3	0	4	4	4
73	CWI 78980	4	4	3	4	4	3	4	4	4	4
74	CWI 78983	4	4	4	4	4	3	3	4	4	4
75	CWI 78984	4	4	4	4	4	4	3	4	4	4
76	CWI 78986	4	4	4	4	4	3	3	4	4	4
77	CWI 78989	4	4	4	4	4	3	0	4	4	4
78	CWI 78990	4	4	4	4	4	4	4	4	4	4
79	CWI 78991	3	4	4	3	4	3	3	4	3	3
80	CWI 80531	4	4	4	3	4	4	4	3	2	4
81	CWI 93151	4	4	4	4	4	4	4	4	3	4
82	CWI 4666	2	1	1	2	1	2	2	1	1	1
83	CWI 4643	0	1	1	1	2	1	0	2	1	1

Infection type (IT) is described as per Si et al. (1992). A 0–4 scale was used to score infection types: 0, 1, and 2 were regarded as resistant phenotypes, and 3 and 4 as susceptible phenotypes.

### Phenotype identification against *Bgt* isolates at the adult stage

The 137 wheat relatives were sown in the field at the adult stage from 2021 to 2023 to determine the resistance to powdery mildew of the mixture of *Bgt* isolates E09, F01, F06, F19, F21, F26, F28, E17, E18, E21, and E23-1. The susceptible control Tainong 18 was highly susceptible in the two cropping seasons. The results demonstrated that most of the tested accessions showed resistance to the powdery mildew, including 103 that were highly resistant and 13 moderately resistant. The remaining 21 wheat relatives were susceptible (Supplementary Table S1). Of these, most of the *T. timopheevii* Zhuk., *T. aestivum* subsp*. spelta*, and *Ae. speltoides* were resistant, suggesting that they were valuable in wheat disease resistance breeding.

### Molecular marker identification

**FIGURE 3:**
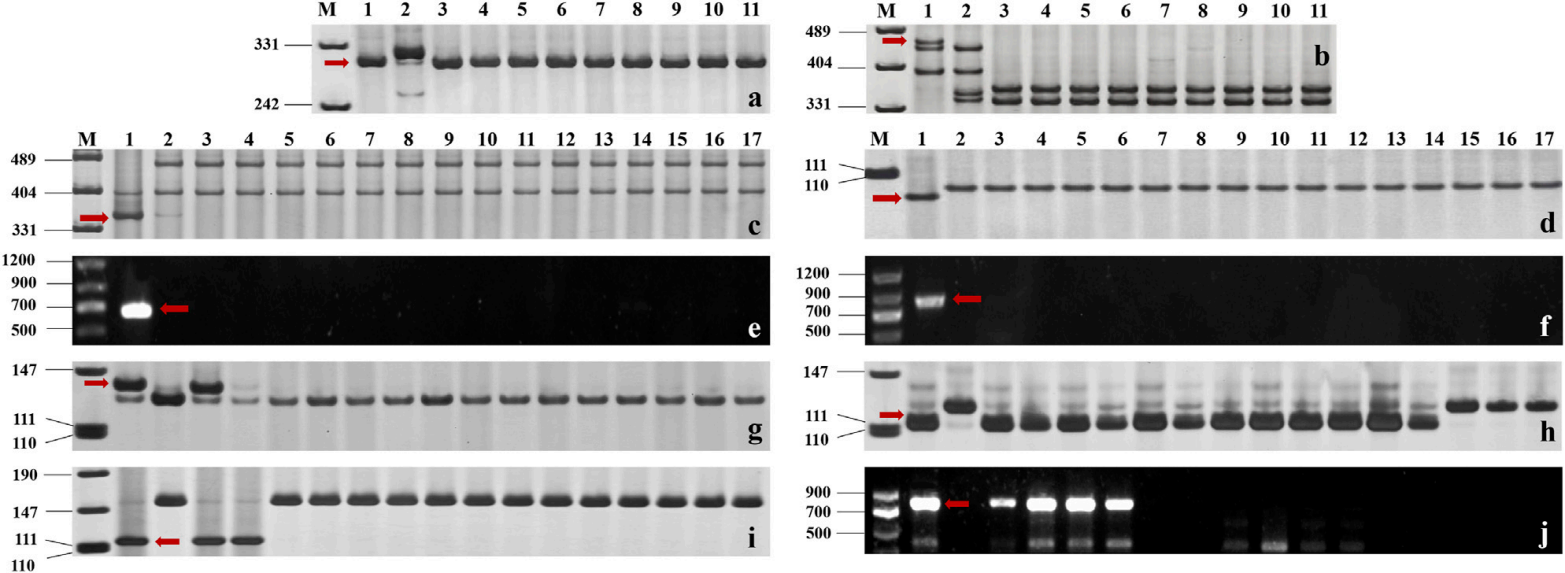
**(A, B)** Amplification patterns of specific markers *CIT02g-18* for *Pm6* **(A)** and *Pm37-82* for *Pm37* **(B)** in part of *T. timopheevii* Zhuk. M: pUC19/Msp I; Lane 1: Coker747 **(A)** and 66 **(B)**; Lane 2: ChineseSpring; and Lanes 3–11: CWI 80427, CWI 80540, CWI 17007, CWI 17224, CWI 17259, CWI 17281, CWI 18175, CWI 18564, and CWI 18594. **(C–F)** Amplification patterns of specific markers *MBH1* for *Pm12* **(C)**, *STS-Pm24* for *Pm24* **(D)**, *sfr43* for *Pm8* **(E)**, and *Pm60-S1* for *Pm60* **(F)** in part of *T. monococcum* L. M: pUC19/*Msp* I **(C–D)**, DNA marker DL1200 **(E–F)**; Lane 1: Yangmai 5 **(C)**, Chiyacao **(D)**, Disponent **(E)**, and EK516 **(F)**; Lane 2: ChineseSpring; and Lanes 3–17: CWI 96237, CWI 96263, CWI 96272, CWI 96275, CWI 17058, CWI 17154, CWI 18949, CWI 19498, CWI 19529, CWI 4393, CWI 4416, CWI 5110, CWI 93034, CWI 93289, and CWI 94083. **(G–J)**: Amplification patterns of specific markers *Pm2b-map-3* for *Pm2* **(G)**, *Xsts24035* for *Pm58* **(H)**, *Xdw12* for *Pm68* **(I)**, and J*S717/718* for *Pm4* **(J)** in part of *T. aestivum* subsp. *spelta*. M: pUC19/Msp I **(G–J)**, DNA marker DL1200 **(J)**; Lane 1: KM2939 **(G)**, AY40 **(H)**, TRI 1796 **(I)**, and VPM1 **(J)**; Lane 2: ChineseSpring; Lanes 3–17 **(G)**: CWI 18468, CWI 14929, CWI 17063, CWI 17247, CWI 17324, CWI 17370, CWI 17677, CWI 17750, CWI 17879, CWI 17892, CWI 17942, CWI 17942, CWI 17954, CWI 17959, and CWI 17964; Lanes 3–17 **(H)**: CWI 17370, CWI 18434, CWI 18439, CWI 18475, CWI 18476, CWI 44154, CWI 44215, CWI 44218, CWI 44409, CWI 80462, CWI 80503, CWI 92915, CWI 96275, CWI 96277, and CWI 96279; Lanes 3–17 **(I)**: CWI 18434, CWI 18439, CWI 14929, CWI 17063, CWI 17247, CWI 17324, CWI 17370, CWI 17677, CWI 17750, CWI 17879, CWI 17892, CWI 17942, CWI 17942, CWI 17954, and CWI 17959; and Lanes 3–17 **(J)**: CWI 18477, CWI 78977, CWI 78976, CWI 80531, CWI 14929, CWI 17063, CWI 17247, CWI 17324, CWI 17370, CWI 17677, CWI 17750, CWI 17879, CWI 17892, CWI 17942, and CWI 17942. Red arrows indicate specific bands.

The functional markers of 12 cloned *Pm* genes *Pm1*-*5*, *Pm8*, *Pm12*, *Pm21*, *Pm24*, *Pm41*, *Pm60,* and *Pm69*, and closely linked or co-segregated markers of the 11 other *Pm* genes *Pm6*, *Pm35*, *Pm37*, *Pm42*, *Pm45*, *Pm47*, *Pm52*, *Pm58*, *Pm59*, *Pm61,* and *Pm68* were used to test 137 wheat relatives to detect the absence/presence of their alleles. The results showed that *Pm58* was detected in 12 *T. aestivum* subsp*. Spelta* with the highest frequency, including CWI 17370, CWI 18434, CWI 18439, CWI 18475, CWI 18476, CWI 44154, CWI 44215, CWI 44218, CWI 44409, CWI 80462, CWI 80503, and CWI 92915. *Pm6*, which was introgressed into common wheat from chromosome 2G of *T. timopheevii*, was detected in 9 *T. timopheevii* Zhuk. *Pm4*, *Pm68,* and *Pm2* were detected in 4, 2, and 1 *T. aestivum* subsp*. spelta* accession, respectively (Figure 2B, Supplementary Table S3). The remaining markers did not produce specific fragments in any of the 137 wheat relatives, suggesting that these accessions most likely did not carry these genes (Figure 3). Notably, all these markers did not produce specific target bands in 109 out of 137 tested wheat accessions, suggesting that these accessions might carry other novel genes or gene combinations (*Pm?*) (Figure 2B).

### Homologous cloning and sequence analysis

**FIGURE 4:**
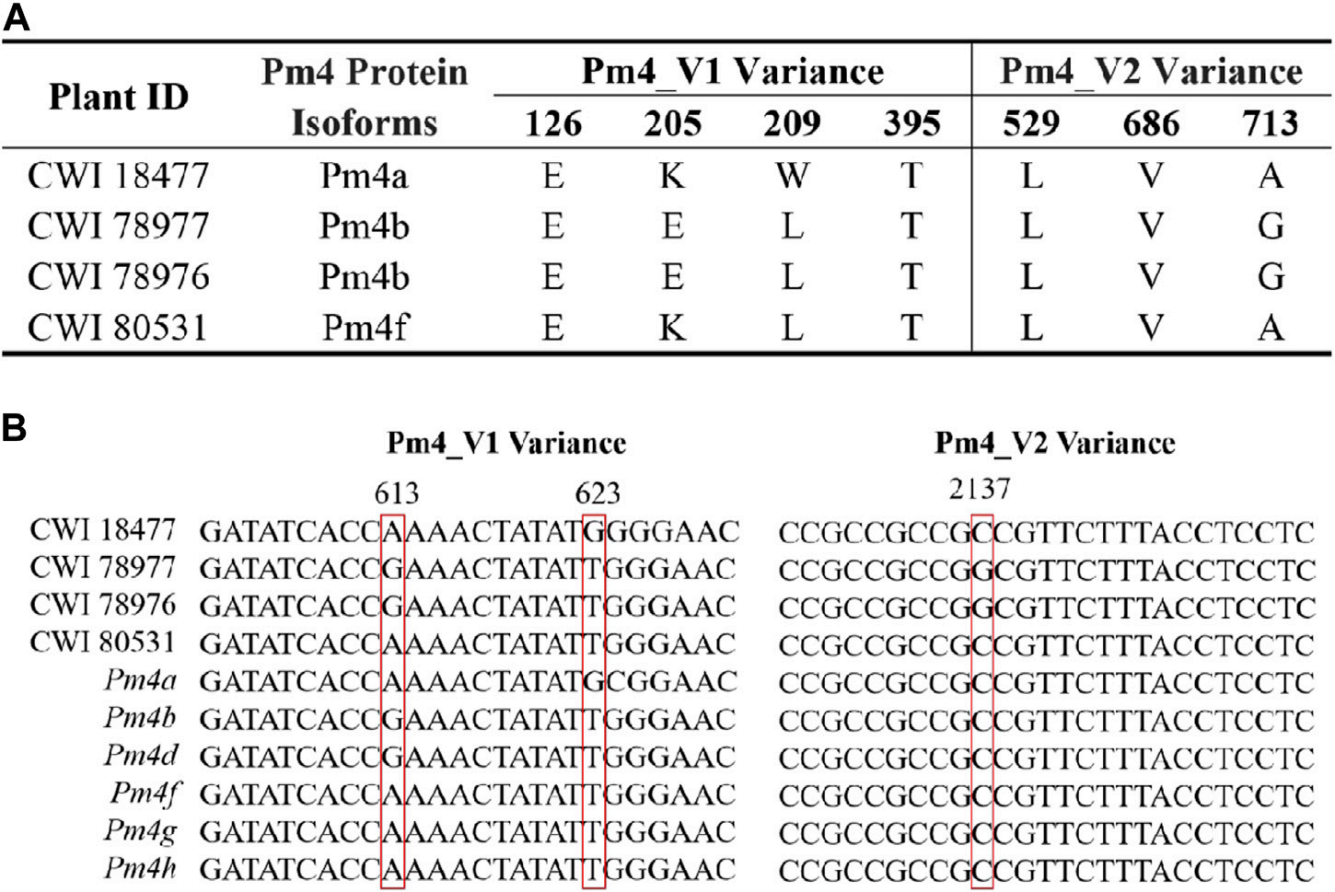
Protein sequence comparison of the *Pm4* variants **(A)** and sequence alignments of CWI 1877, CWI 78977, CWI 78976, CWI 80531, and *Pm4* alleles **(B)**. Red box indicates nucleotide variant sites.

**FIGURE 5:**
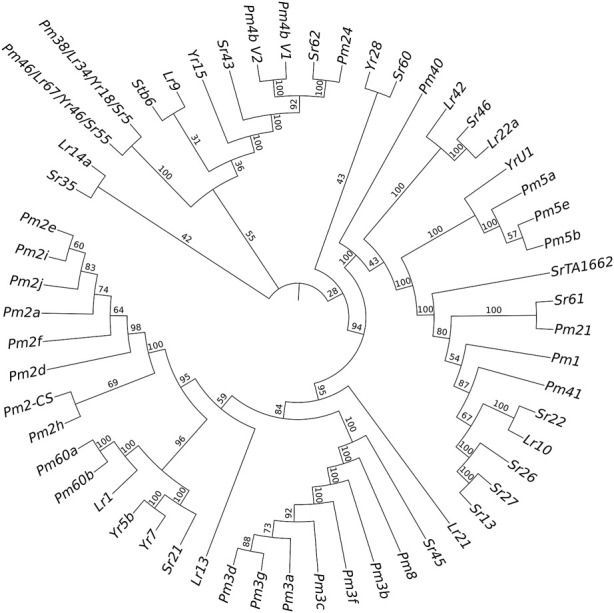
Phylogenetic tree construction results of powdery mildew (*Pm*), stem rust (*Sr*), leaf rust (*Lr*), and yellow rust (*Yr*) resistant genes.

Only the cloned genes *Pm2* and *Pm4* were detected in the marker detection. Previous studies reported that *Pm2* in the released rartuidy wheat cultivars were all of the *Pm2a* haplotype (Yu et al., 2022). Therefore, we focus on the haplotype identification of *Pm4* locus in this study. Using the nest PCR, we cloned the *Pm4* alleles from the four putative *Pm4*-carrying *T*. subsp*. spelta* accessions. Sequence alignment suggested that CWI 18477 carried *Pm4a*, CWI 78976 and CWI 78977 carried *Pm4b*, and CWI 80531 carried *Pm4f* (Figure 4). The alleles of *Pm4c*, *Pm4d*, *Pm4e*, *Pm4g*, and *Pm4h* were not detected. Additionally, comparison of the *Pm4* CDS sequence with a series of 54 cloned disease-resistant genes showed that *Pm4* was not related to the NLR genes but close to *Pm24* and *Sr62*, which included tandem kinase domain (Figure 5).

**FIGURE 6:**
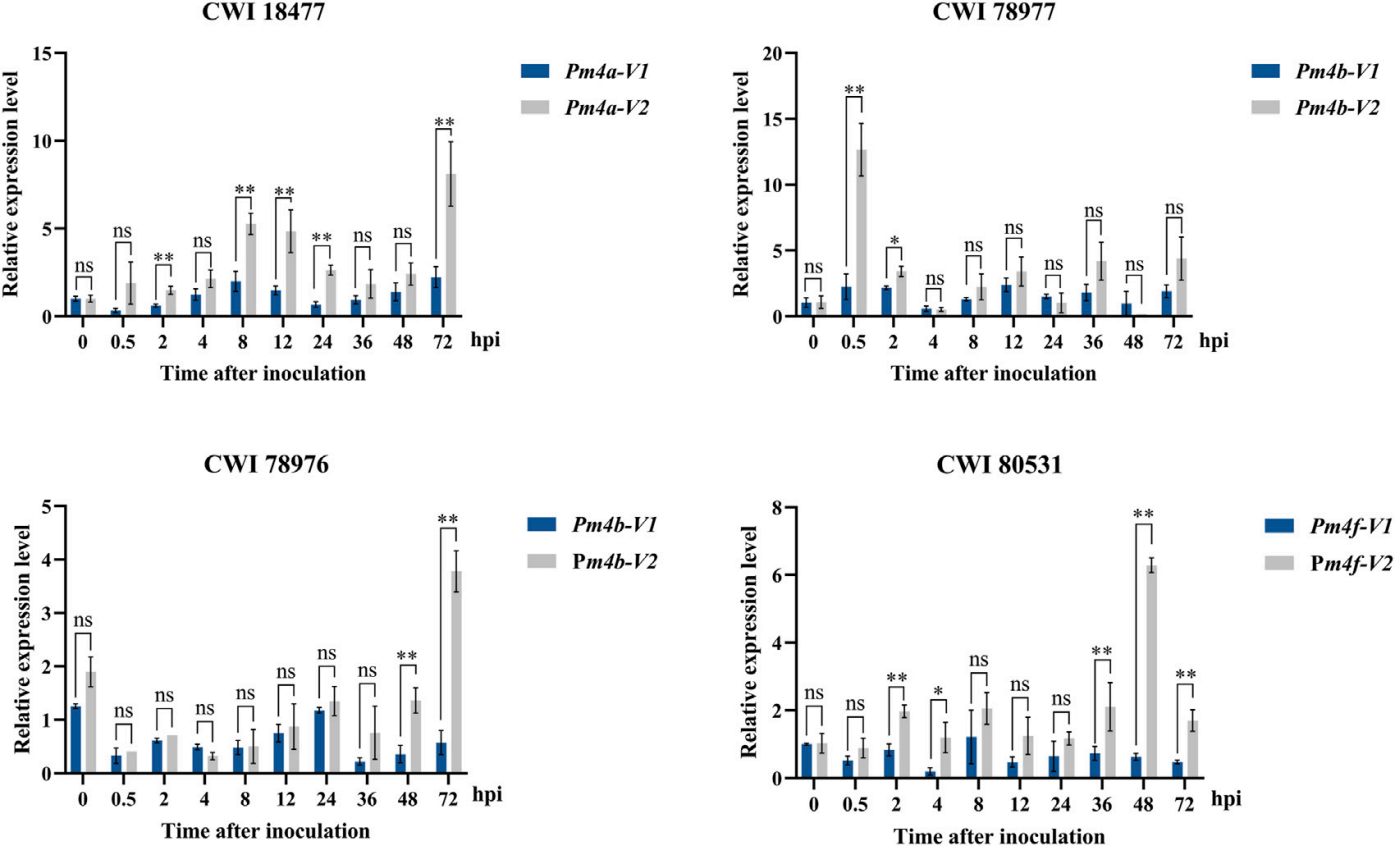
Expression patterns of the *Pm4-V1* and *Pm4-V2* splice variants in wheat CWI 18477, CWI 78977, CWI 78976, and CWI 80531 during a 72-h time course. Error bars represent SD based on three biological replicates. Statistical analysis used a *t*-test at *p* < 0.05 (*p < 0.05, **p < 0.01, ns: not significant). TaActin was used as the internal control.

**Expression pattern analysis of *Pm4* alleles.** To identify the relationship between powdery mildew resistance and gene transcription levels, we used qRT-PCR to monitor the expression patterns of *Pm4* alleles in those four putative *Pm4*-carrying *T. aestivum* subsp*. spelta* accessions after inoculating with *Bgt* isolate E09 at different times. As in Figure 6, the expression levels of all *Pm4* alleles were significantly upregulated after inoculation. It is supposed that *Pm4* alleles played a key role in disease resistance in these wheat genetic backgrounds. In addition, no significant differences between *Pm4-V1* and *Pm4-V2* were observed most of the time after inoculation, suggesting both two transcript splices were important.

## Discussion

Wheat contributes to the diet of approximately 35% of the world’s population and is of vital importance in guaranteeing global food security. Powdery mildew threatens wheat yield and quality. Fortunately, some common wheat accessions and their relatives carry genes that allow resistance to powdery mildew during the growing period. The narrow genetic basis of common wheat has expanded the demand for diversified germplasm resources against powdery mildew. In general, the genes that were derived from wild relatives of common wheat cannot be directly applied to wheat breeding due to the undesirable linkage drags or poor agronomic traits of the donors, such as rye (*S. cereale* L, 2n = 2x = 14, RR) and *Agropyron cristatum* (2n = 2x = 14, VV) (Zhu et al., 2023). By comparison, the secondary gene bank of common wheat is an important gene donor in the genetic improvement of common wheat because of its abundant genetic variation and close kinship with wheat. They thus make it easier to balance disease resistance and comprehensive agronomic traits. In this study, a series of 137 wheat relatives, including 53 *T. monococcum* L., 6 *T. rartu Thumanjan ex* Gandilyan, 9 *T. timopheevii* Zhuk., 66 *T. aestivum* subsp*. spelta*, and 3 *Ae. Speltoides*, were systematically evaluated for their powdery mildew resistance and composition of *Pm* genes.

*T. monococcum* L. and *T. rartu Thumanjan ex* Gandilyan are diploid wild ancestral species of common wheat. They are also valuable germplasms for wheat improvement. Previous studies reported that *T. rartu* contained more abundant nucleotide-binding and leucine-rich repeat domain (NB-LRR) genes than maize, sorghum, or rice, making it a good source for *Pm* resistance genes (Ling et al., 2013). Zou et al. (2018) identified a new *Pm* gene, *Pm60,* from the resistant *T. urartu* variety PI428309 using the strategies of genetic mapping and RNA-sequencing. Another study also showed that a *Pm*-resistant locus in *T. urartu* was mapped to chromosome arm 7AL in a similar position to *Pm60* (Qiu et al., 2005). Additionally, *T. monococcum* L. is also an important donor of *Pm* genes. Several *Pm* genes, such as *Pm1b* (Hu et al., 1997), *Mlm80* (Yao et al., 2007), *Mlm 2033* (Yao et al., 2007), *pm 2026* (Xu et al., 2008), *Pm4d* (Schmolke et al., 2012), and *Pm25* (Shi et al., 1998), were identified in *T. monococcum* L. In our study, 34 out of 53 *T. monococcum* L. (64.15%) and five of six *T. urartu Thumanjan ex* Gandilyan (83.33%) were resistant to *Bgt* isolate E09 at the seedling stage. Fortunately, 52 accessions were resistant to the mixture *Bgt* isolates at the adult stage. After marker detection, all 57 accessions carried none of the 23 tested *Pm* genes. We speculate that there may be other unknown *Pm* genes in these accessions, which provide potential new *Pm* genes to be mined.

*Ae*. *speltoides* is the closest extant relative of the wheat B subgenome which provides resistance to multiple diseases (Luo et al., 2005). To date, *Pm12*, *Pm32*, and *Pm53* derived from *Ae*. *speltoides* have also been introduced into the wheat genetic background. In our study, *Ae*. *speltoides* accessions CWI 4643 and CWI 4666 from Iraq were resistant to all ten *Bgt* isolates but did not carry any of the tested *Pm* genes, indicating their potential value in resistance breeding for wheat. Additionally, *Ae. speltoides* has also served as a resistance source against leaf rust, including *Lr28*, *Lr35*, *Lr36*, *Lr47*, and *Lr51* (Helguera et al., 2005), and stem rust, including *Sr32*, *Sr39*, *Sr47*, and *SrAes7t* (Klindworth et al., 2012). More than 200 *Ae*. *speltoides* accessions collected from five areas of Israel showed high resistance to stem rust, leaf rust, and stripe rust in both Israel and Minnesota, USA (Anikster et al., 2005). It is therefore likely that additional genes for disease resistance for use in wheat breeding could be found in *Ae*. *speltoides*.

*Pm4* is an important *Pm* gene that has already been widely distributed across parts of China and other countries because of its resistance to *Bgt* isolates (Wang et al., 2005). Eight alleles (*Pm4a–Pm4h*) at the *Pm4* locus have been successively reported (Sánchez-Martín et al., 2021). Sánchez-Martín et al. (2021) recently cloned *Pm4b* using the MutChromSeq strategy. The functional marker *JS717/JS718* was designed and could be used to diagnose *Pm4* alleles. In our study, *Pm4* was detected in 4 *T. aestivum* subsp*. spelta* accessions. After homologous cloning and sequence alignment, CWI 18477 carried *Pm4a*, CWI 78976 and CWI 78977 carried *Pm4b*, and CWI 80531 carried *Pm4f*. The alleles of *Pm4c*, *Pm4d*, *Pm4e*, *Pm4g*, and *Pm4h* were not detected. Curiously, CWI 78976 and CWI 78977 carried *Pm4b* but exhibited contrasting levels of resistance to *Bgt* isolate E09. This cannot be explained solely by the differences in genetic backgrounds; there may be some other unknown *Pm* genes interacting with each other to provide potential resistance.

Notably, the diagnostic markers of 23 tested *Pm* genes did not produce specific target bands in 109 of 137 tested wheat accessions, suggesting that these accessions might carry other novel genes or gene combinations. There is therefore an urgent need to further analyze the resistant gene(s) in those accessions that did not carry tested *Pm* gene(s) by fine mapping and other technologies. Except for *Pm* genes, wheat relatives contain abundant yield, stress resistance, and resistant genes. Therefore, these germplasm resources can be better applied in wheat resistance breeding (Wang et al., 2023).

In summary, this study evaluated the powdery mildew resistance and composition of *Pm* genes of 137 wheat relatives. The results provided reference information and germplasms for wheat improvement against powdery mildew.

## Conclusion

A total of 137 wheat relatives, including 53 *T*. *monococcum*, 6 *Triticum urartu*, 9 *T. timopheevii* Zhuk., 66 *T*. *aestivum* subsp*. spelta*, and 3 *Ae*. *speltoides*, were systematically evaluated their powdery mildew resistance and composition of *Pm* genes. Our study provided reference information and abundant gene resources for wheat resistance molecular breeding.

## Data Availability

The original contributions presented in the study are included in the article/Supplementary Material; further inquiries can be directed to the corresponding authors.
