# Supplementary material for: Evaluation and identification of powdery mildew-resistant genes in 137 wheat relatives
Source: Front Genet. 2024 Jan 24;15:1342239. doi: 10.3389/fgene.2024.1342239 (PMC10847533; doi:10.3389/fgene.2024.1342239)
Supplement: Supplementary file 1 [file Table1.docx]

**Table S1 The list of wheat relatives in this study and infection type**

| **No.** | **Plant ID** | **Plant Name** | **Genomes** | **Taxonomy** | **Origin** | **Infection type** | |
| --- | --- | --- | --- | --- | --- | --- | --- |
|  |  |  |  |  |  | **Seeding *Bgt* E09** | **Adult stage** |
| 1 | CWI 80465 | T.MONOCOCCUM PI167591 | AA | *Triticum monococcum* | United States | 4 | MS |
| 2 | CWI 80556 | GEO001 1 514 | AA | *Triticum monococcum* | Georgia | 4 | HR |
| 3 | CWI 83497 | GEO001 1 4 | AA | *Triticum monococcum* | Georgia | 0 | HR |
| 4 | CWI 96237 | RCAT 003585 | AA | *Triticum monococcum* | France | 0 | HR |
| 5 | CWI 96263 | BGRC 43463 | AA | *Triticum monococcum* | Turkey | 0 | HR |
| 6 | CWI 96272 | BGRC 43481 | AA | *Triticum monococcum* | Greece | 0 | HR |
| 7 | CWI 96275 | BGRC 43483 | AA | *Triticum monococcum* | Greece | 0 | HR |
| 8 | CWI 96277 | BGRC 43485 | AA | *Triticum monococcum* | Greece | 0 | HR |
| 9 | CWI 96279 | BGRC 43487 | AA | *Triticum monococcum* | Bulgaria | 0 | HR |
| 10 | CWI 96281 | GID 7255529 | AA | *Triticum monococcum* | Bulgaria | 0 | HR |
| 11 | CWI 96303 | BGRC 42017 | AA | *Triticum monococcum* | Albania | 4 | HR |
| 12 | CWI 96353 | ALB002-7224 | AA | *Triticum monococcum* | Albania | 1 | HR |
| 13 | CWI 16957 | T.MONOCOCCUM PI94740 | AA | *Triticum monococcum subsp. monococcum* | Spain | 3 | MS |
| 14 | CWI 16959 | T.MONOCOCCUM PI94743 | AA | *Triticum monococcum subsp. monococcum* | Russian Federation | 4 | MS |
| 15 | CWI 17058 | T.MONOCOCCUM PI221413 | AA | *Triticum monococcum subsp. monococcum* | Serbia | 0 | HR |
| 16 | CWI 17154 | T.MONOCOCCUM PI272562 | AA | *Triticum monococcum subsp. monococcum* | Hungary | 0 | HR |
| 17 | CWI 18949 | T.MONOCOCCUM PI428155 | AA | *Triticum monococcum subsp. monococcum* | United Kingdom | 3 | HR |
| 18 | CWI 19498 | T.MONOCOCCUM RL5455 | AA | *Triticum monococcum subsp. monococcum* | Turkey | 0 | HR |
| 19 | CWI 19529 | T.MONOCOCCUM RL5509 | AA | *Triticum monococcum subsp. monococcum* | Iran | 4 | HR |
| 20 | CWI 19531 | T.MONOCOCCUM RL5511 | AA | *Triticum monococcum subsp. monococcum* | Iran | 4 | HR |
| 21 | CWI 19535 | T.MONOCOCCUM RL5515 | AA | *Triticum monococcum subsp. monococcum* | Iran | 0 | HR |
| 22 | CWI 19536 | T.MONOCOCCUM RL5516 | AA | *Triticum monococcum subsp. monococcum* | Iran | 4 | HR |
| 23 | CWI 2352 | TR39426 | AA | *Triticum monococcum subsp. monococcum* | Turkey | 1 | HR |
| 24 | CWI 38331 | BG 13629 | AA | *Triticum monococcum* subsp*. monococcum* | Spain | 1 | HR |
| 25 | CWI 5103 | T.MO G3300 | AA | *Triticum monococcum* subsp*. monococcum* | Serbia | 2 | HR |
| 26 | CWI 6265 | HARLAN J.R 3094 | AA | *Triticum monococcum* subsp*. monococcum* | Turkey | 0 | HR |
| 27 | CWI 83793 | ALB002-7532 | AA | *Triticum monococcum* subsp*. monococcum* | Albania | 4 | HR |
| 28 | CWI 83803 | ALB002-7542 | AA | *Triticum monococcum* subsp*. monococcum* | Albania | 4 | HR |
| 29 | CWI 17260 | T.BOEOTICUM (75) | AA | *Triticum monococcum* subsp*. aegilopoides* | United States | 0 | HR |
| 30 | CWI 18643 | T.BOEOTICUM (91) | AA | *Triticum monococcum* subsp*. aegilopoides* | United States | 0 | HR |
| 31 | CWI 18754 | T.BOEOTICUM (93) | AA | *Triticum monococcum* subsp*. aegilopoides* | United States | 0 | HR |
| 32 | CWI 18758 | T.BOEOTICUM PI470726 | AA | *Triticum monococcum* subsp*. aegilopoides* | United States | 0 | HR |
| 33 | CWI 4374 | T.BOEOTICUM (31) | AA | *Triticum monococcum* subsp*. aegilopoides* | United States | 3 | HR |
| 34 | CWI 4385 | T.BOEOTICUM (34) | AA | *Triticum monococcum* subsp*. aegilopoides* | United States | 0 | HR |
| 35 | CWI 4393 | T.BOEOTICUM PI306527 | AA | *Triticum monococcum* subsp*. aegilopoides* | Romania | 4 | HS |
| 36 | CWI 4416 | T.BOEOTICUM G1781 | AA | *Triticum monococcum* subsp*. aegilopoides* | Turkey | 1 | HR |
| 37 | CWI 5110 | PI 542475 | AA | *Triticum monococcum* subsp*. aegilopoides* | United States | 0 | HR |
| 38 | CWI 93034 | T.BOEOTICUM (3) | AA | *Triticum monococcum* subsp*. aegilopoides* | United States | 0 | HR |
| 39 | CWI 93289 | T.BOEOTICUM (27) | AA | *Triticum monococcum* subsp*. aegilopoides* | United States | 4 | HR |
| 40 | CWI 94083 | T.BOEOTICUM (29) | AA | *Triticum monococcum* subsp*. aegilopoides* | United States | 0 | HR |
| 41 | CWI 94105 | T.BOEOTICUM (37) | AA | *Triticum monococcum* subsp*. aegilopoides* | United States | 0 | MS |
| 42 | CWI 94107 | T.BOEOTICUM PI427720 | AA | *Triticum monococcum* subsp*. aegilopoides* | Iraq | 0 | HR |
| 43 | CWI 94109 | T.BOEOTICUM G1757 | AA | *Triticum monococcum* subsp*. aegilopoides* | Armenia | 0 | HR |
| 44 | CWI 94112 | T.BOEOTICUM G1870 | AA | *Triticum monococcum* subsp*. aegilopoides* | Turkey | 3 | HR |
| 45 | CWI 94115 | T.BOEOTICUM G3351 | AA | *Triticum monococcum* subsp*. aegilopoides* | Germany | 0 | HR |
| 46 | CWI 94145 | PI 487249 | AA | *Triticum monococcum* subsp*. aegilopoides* | United States | 0 | HR |
| 47 | CWI 94149 | T.BOEOTICUM PI427504 | AA | *Triticum monococcum* subsp*. aegilopoides* | Turkey | 0 | HR |
| 48 | CWI 94153 | T.BOEOTICUM (67) | AA | *Triticum monococcum* subsp*. aegilopoides* | United States | 4 | HR |
| 49 | CWI 94157 | T.BOEOTICUM (79) | AA | *Triticum monococcum* subsp*. aegilopoides* | United States | 0 | HR |
| 50 | CWI 94169 | T.BOEOTICUM PI427708 | AA | *Triticum monococcum* subsp*. aegilopoides* | Iraq | 3 | HR |
| 51 | CWI 94171 | T.BOEOTICUM PI306526 | AA | *Triticum monococcum* subsp*. aegilopoides* | Romania | 3 | HR |
| 52 | CWI 96319 | T.BOEOTICUM (71) | AA | *Triticum monococcum* subsp*. aegilopoides* | United States | 3 | HR |
| 53 | CWI 17195 | T.BOEOTICUM PI277123 | AA | *Triticum monococcum* subsp*. aegilopoides* | United States | 2 | HR |
| 54 | CWI 52947 | T.URARTU PI427328 | AA | *Triticum urartu* | Iraq | 0 | HR |
| 55 | CWI 19082 | T.URARTU PI428314 | AA | *Triticum urartu* | Lebanon | 0 | MS |
| 56 | CWI 19093 | T.URARTU PI428333 | AA | *Triticum urartu* | Lebanon | 0 | MS |
| 57 | CWI 19047 | T.URARTU PI428269 | AA | *Triticum urartu* | Lebanon | 1 | HR |
| 58 | CWI 19058 | T.URARTU PI428280 | AA | *Triticum urartu* | Lebanon | 1 | HR |
| 59 | CWI 53111 | T.URARTU PI487267 | AA | *Triticum urartu* | Syria | 3 | HR |
| 60 | CWI 80427 | GEO001 1448 | AA | *Triticum timopheevii* | Georgia | 0 | HR |
| 61 | CWI 80540 | GEO001 1621 | AA | *Triticum timopheevii* | Georgia | 4 | MS |
| 62 | CWI 17007 | T.TIMOPHEEVII PI190974 | AAGG | *Triticum timopheevii* subsp*. timopheevii* | Spain | 0 | HR |
| 63 | CWI 17224 | T.TIMOPHEEVII PI288033 | AAGG | *Triticum timopheevii* subsp*. timopheevii* | Australia | 0 | HR |
| 64 | CWI 17259 | T.DICOCCOIDES PI300991 | AAGG | *Triticum timopheevii* subsp*. timopheevii* | Israel | 0 | HR |
| 65 | CWI 17281 | T.TIMOPHEEVII PI306557 | AAGG | *Triticum timopheevii* subsp*. timopheevii* | Romania | 0 | HR |
| 66 | CWI 18175 | T.TIMOPHEEVII PI349054 | AAGG | *Triticum timopheevii* subsp*. timopheevii* | Georgia | 0 | HR |
| 67 | CWI 18564 | T.ARARATICUM PI427373 | AAGG | *Triticum timopheevii* subsp*. armeniacum* | Iran | 0 | HR |
| 68 | CWI 18594 | T.ARARATICUM PI427429 | AAGG | *Triticum timopheevii* subsp*. armeniacum* | Iraq | 0 | HR |
| 69 | CWI 14929 | KLEIN CONDOR | AABBDD | *Triticum aestivum* subsp*. spelta* | United States | 4 | MS |
| 70 | CWI 17063 | T.SPELTA PI221420 | AABBDD | *Triticum aestivum* subsp*. spelta* | Serbia | 0 | HR |
| 71 | CWI 17247 | T.SPELTA PI295068 | AABBDD | *Triticum aestivum* subsp*. spelta* | Bulgaria | 0 | HR |
| 72 | CWI 17324 | T.SPELTA PI347864 | AABBDD | *Triticum aestivum* subsp*. spelta* | Switzerland | 4 | HR |
| 73 | CWI 17370 | T.SPELTA PI347914 | AABBDD | *Triticum aestivum* subsp*. spelta* | Switzerland | 4 | HS |
| 74 | CWI 17677 | T.SPELTA PI348235 | AABBDD | *Triticum aestivum* subsp*. spelta* | Germany | 0 | HR |
| 75 | CWI 17750 | T.SPELTA PI348317 | AABBDD | *Triticum aestivum* subsp*. spelta* | Belgium | 0 | HR |
| 76 | CWI 17879 | T.SPELTA PI348465 | AABBDD | *Triticum aestivum* subsp*. spelta* | Spain | 1 | HR |
| 77 | CWI 17892 | T.SPELTA PI348478 | AABBDD | *Triticum aestivum* subsp*. spelta* | Spain | 0 | MS |
| 78 | CWI 17942 | T.SPELTA PI348531 | AABBDD | *Triticum aestivum* subsp*. spelta* | Spain | 3 | HR |
| 79 | CWI 17954 | T.SPELTA PI348544 | AABBDD | *Triticum aestivum* subsp*. spelta* | Spain | 0 | HR |
| 80 | CWI 17959 | T.SPELTA PI348549 | AABBDD | *Triticum aestivum* subsp*. spelta* | Spain | 0 | HR |
| 81 | CWI 17964 | T.SPELTA PI348554 | AABBDD | *Triticum aestivum* subsp*. spelta* | Spain | 1 | MR |
| 82 | CWI 18042 | T.SPELTA PI348638 | AABBDD | *Triticum aestivum* subsp*. spelta* | Spain | 4 | MR |
| 83 | CWI 18043 | T.SPELTA PI348639 | AABBDD | *Triticum aestivum* subsp*. spelta* | Spain | 3 | MR |
| 84 | CWI 18065 | T.SPELTA PI348661 | AABBDD | *Triticum aestivum* subsp*. spelta* | Spain | 1 | HR |
| 85 | CWI 18067 | T.SPELTA PI348663 | AABBDD | *Triticum aestivum* subsp*. spelta* | Spain | 4 | MR |
| 86 | CWI 18080 | T.SPELTA PI348676 | AABBDD | *Triticum aestivum* subsp*. spelta* | Spain | 0 | HR |
| 87 | CWI 18090 | T.SPELTA PI348688 | AABBDD | *Triticum aestivum* subsp*. spelta* | Spain | 3 | MR |
| 88 | CWI 18143 | T.SPELTA PI348748 | AABBDD | *Triticum aestivum* subsp*. spelta* | Spain | 0 | HR |
| 89 | CWI 18165 | T.SPELTA PI348775 | AABBDD | *Triticum aestivum* subsp*. spelta* | Spain | 0 | MR |
| 90 | CWI 18166 | T.SPELTA PI348776 | AABBDD | *Triticum aestivum* subsp*. spelta* | Spain | 0 | HR |
| 91 | CWI 18353 | T.SPELTA PI355600 | AABBDD | *Triticum aestivum* subsp*. spelta* | Switzerland | 0 | MR |
| 92 | CWI 18371 | T.SPELTA PI355618 | AABBDD | *Triticum aestivum* subsp*. spelta* | Belgium | 1 | HR |
| 93 | CWI 18434 | T.SPELTA PI355681 | AABBDD | *Triticum aestivum* subsp*. spelta* | Belgium | 0 | HR |
| 94 | CWI 18439 | T.SPELTA PI355686 | AABBDD | *Triticum aestivum* subsp*. spelta* | Germany | 4 | HR |
| 95 | CWI 18453 | T.SPELTA PI355700 | AABBDD | *Triticum aestivum* subsp*. spelta* | Germany | 4 | MR |
| 96 | CWI 18468 | T.SPELTA PI362062 | AABBDD | *Triticum aestivum* subsp*. spelta* | Romania | 4 | HR |
| 97 | CWI 18475 | T.SPELTA PI367201 | AABBDD | *Triticum aestivum* subsp*. spelta* | Afghanistan | 4 | HS |
| 98 | CWI 18476 | T.SPELTA PI367202 | AABBDD | *Triticum aestivum* subsp*. spelta* | Afghanistan | 4 | HS |
| 99 | CWI 18477 | T.SPELTA PI367203 | AABBDD | *Triticum aestivum* subsp*. spelta* | Afghanistan | 4 | HR |
| 100 | CWI 19131 | T.SPELTA CI2968 | AABBDD | *Triticum aestivum* subsp*. spelta* | United States | 0 | HR |
| 101 | CWI 44151 | T.SPELTA 001515 | AABBDD | *Triticum aestivum* subsp*. spelta* | Spain | 4 | HR |
| 102 | CWI 44154 | T.SPELTA 001521 | AABBDD | *Triticum aestivum* subsp*. spelta* | Germany | 0 | HR |
| 103 | CWI 44183 | T.SPELTA 001552 | AABBDD | *Triticum aestivum* subsp*. spelta* | Russian Federation | 4 | HS |
| 104 | CWI 44215 | T.SPELTA 001585 | AABBDD | *Triticum aestivum* subsp*. spelta* | Iran | 4 | HS |
| 105 | CWI 44218 | T.SPELTA 001589 | AABBDD | *Triticum aestivum* subsp*. spelta* | Iran | 4 | HS |
| 106 | CWI 44355 | T.SPELTA 013293 | AABBDD | *Triticum aestivum* subsp*. spelta* | Italy | 0 | HR |
| 107 | CWI 44394 | T.SPELTA 013488 | AABBDD | *Triticum aestivum* subsp*. spelta* | Switzerland | 4 | MS |
| 108 | CWI 44396 | T.SPELTA 013490 | AABBDD | *Triticum aestivum* subsp*. spelta* | Switzerland | 0 | HR |
| 109 | CWI 44398 | T.SPELTA 013493 | AABBDD | *Triticum aestivum* subsp*. spelta* | Switzerland | 4 | HR |
| 110 | CWI 44403 | T.SPELTA 013498 | AABBDD | *Triticum aestivum* subsp*. spelta* | Switzerland | 0 | HR |
| 111 | CWI 44409 | T.SPELTA 013504 | AABBDD | *Triticum aestivum* subsp*. spelta* | Switzerland | 4 | HR |
| 112 | CWI 44410 | T.SPELTA 013505 | AABBDD | *Triticum aestivum* subsp*. spelta* | Switzerland | 3 | HR |
| 113 | CWI 78968 | BALMEGG | AABBDD | *Triticum aestivum* subsp*. spelta* | Germany | 4 | HR |
| 114 | CWI 78972 | FRANCKENKORN | AABBDD | *Triticum aestivum* subsp*. spelta* | Germany | 0 | HR |
| 115 | CWI 78976 | HUBEL | AABBDD | *Triticum aestivum* subsp*. spelta* | Czech Republic | 3 | HR |
| 116 | CWI 78977 | LUEG | AABBDD | *Triticum aestivum* subsp*. spelta* | Czech Republic | 0 | MR |
| 117 | CWI 78979 | OBERKULMER ROTKORN | AABBDD | *Triticum aestivum* subsp*. spelta* | Czech Republic | 1 | HR |
| 118 | CWI 78980 | OSTRO | AABBDD | *Triticum aestivum* subsp*. spelta* | Czech Republic | 0 | MR |
| 119 | CWI 78981 | REDOUTE | AABBDD | *Triticum aestivum* subsp*. spelta* | Germany | 4 | HR |
| 120 | CWI 78982 | RENVAL | AABBDD | *Triticum aestivum* subsp*. spelta* | Belgium | 4 | HR |
| 121 | CWI 78983 | ROTER SCHLEGELDINKEL | AABBDD | *Triticum aestivum* subsp*. spelta* | Germany | 0 | MR |
| 122 | CWI 78984 | ROUQUIN | AABBDD | *Triticum aestivum* subsp*. spelta* | Germany | 0 | HR |
| 123 | CWI 78985 | SCHWABENKORN | AABBDD | *Triticum aestivum* subsp*. spelta* | Germany | 4 | HR |
| 124 | CWI 78986 | SCHWABENSPELZ | AABBDD | *Triticum aestivum* subsp*. spelta* | Germany | 0 | MR |
| 125 | CWI 78989 | VOGELS DINKEL | AABBDD | *Triticum aestivum* subsp*. spelta* | Germany | 0 | HR |
| 126 | CWI 78990 | WAGGERSHAUSERS H.W.K | AABBDD | *Triticum aestivum* subsp*. spelta* | Germany | 0 | HR |
| 127 | CWI 78991 | WEISSER-KOLBENSPELZ | AABBDD | *Triticum aestivum* subsp*. spelta* | Germany | 0 | HR |
| 128 | CWI 80462 | GEO001 1511 | AABBDD | *Triticum aestivum* subsp*. spelta* | Georgia | 4 | HR |
| 129 | CWI 80503 | GEO001 1574 | AABBDD | *Triticum aestivum* subsp*. spelta* | Georgia | 4 | HR |
| 130 | CWI 80504 | GEO001 1575 | AABBDD | *Triticum aestivum* subsp*. spelta* | Georgia | 4 | HS |
| 131 | CWI 80531 | GEO001 1606 | AABBDD | *Triticum aestivum* subsp*. spelta* | Georgia | 0 | MR |
| 132 | CWI 87084 | WIR 52464 | AABBDD | *Triticum aestivum* subsp*. spelta* | Tajikistan | 4 | HS |
| 133 | CWI 92915 | PI 469032 | AABBDD | *Triticum aestivum* subsp*. spelta* | United States | 4 | HS |
| 134 | CWI 93151 | PI 520066 | AABBDD | *Triticum aestivum* subsp*. spelta* | United States | 0 | MS |
| 135 | CWI 4666 | AE.SPELTOIDES G2333 | SS | *Aegilops speltoides* | Iraq | 0 | HR |
| 136 | CWI 4643 | AE.SPELTOIDES G2297 | SS | *Aegilops speltoides* | Iraq | 0 | HR |
| 137 | CWI 48188 | AE.SPELTOIDES PI542252 | SS | *Aegilops speltoides* | Turkey | 4 | HR |

Infection type (IT) described as Si et al., (1992). 0–4 scale was used to score the infection types: 0, 0;, 1 and 2 were regarded as resistant phenotypes, 3 and 4 were susceptible phenotypes.

**Table S2 The list of markers in this study**

| **Gene** | **Positive control** | **Primer** | **Primer sequence (5’-3’)** | **Size(bp)** | **Gel type** | **Molecularmarker type** | **References** |
| --- | --- | --- | --- | --- | --- | --- | --- |
| *Pm1* | Axminster/8*Cc | *Pm1aSTS1-F* | CAATATAAACTTCAGATGTTCTATTCTCAAAC | 333bp | Agarose | STS | Hewitt et al., 2021 |
|  |  | *Pm1aSTS1-R* | CTACATTGGCTATGCGTGTAGTC |  |  |  |  |
| *Pm2* | KM2939 | *Pm2b-map-3-F* | TGGTAACGAAGGTTGTCGCC | 150bp | PAGE | InDel | Jin et al., 2021 |
|  |  | *Pm2b-map-3-R* | GCTCAATCTGAGAACCTT |  |  |  |  |
| *Pm3* | Asosan | *Pm3a-F* | GGAGTCTCTTCGCATAGA | - | PAGE | SSR | Tommasini et al., 2006 |
|  |  | *Pm3a-R* | CAGCTTCTAAGATCAAGGAT |  |  |  |  |
|  | Chul | *Pm3b-F* | GGCACAGACAAAGCTCTG | - | PAGE | SSR |  |
|  |  | *Pm3b-R* | TCGAGTAGCTCGGGAATC |  |  |  |  |
|  | Sonora | *Pm3c-F* | CTAGTGGAGGTAGTTGAC | - | PAGE | SSR |  |
|  |  | *Pm3c-R* | AGTCGTTCAAGAGAACGGC |  |  |  |  |
|  | Kolibri | *Pm3d-F* | TGACTATTCGTGGGTGCA | - | PAGE | SSR |  |
|  |  | *Pm3d-R* | GACTGCGGCACAGTTCAGC |  |  |  |  |
|  | W150 | *Pm3e-F* | GGAATCCCTTTGGCTTGT | - | PAGE | SSR |  |
|  |  | *Pm3e-R* | CTAGCAGAGCAGTGCAAG |  |  |  |  |
|  | Michigan Amber | *Pm3f-F* | GGAGTCTCTTTGCTTAAG | - | PAGE | SSR |  |
|  |  | *Pm3f-R* | CAGCTTCTAAGATCAAGGAT |  |  |  |  |
|  | Aristide | *Pm3g-F* | GAATCCCTTTATCTTGAC | - | PAGE | SSR |  |
|  |  | *Pm3g-R* | ATTCCCCTAGCAGAGCAGAA |  |  |  |  |
| *Pm4* | VPM1 | *JS717* | AGGTGGACATCCTAGGCGCT | 926bp | Agarose | SSR | Sánchez-Martín et al., 2021 |
|  |  | *JS718* | GATCTGGGTACCACAGCACCG |  |  |  |  |
| *Pm5* | Fuzhuang30 | *Pm5e KASP-F1* | GAAGGTGACCAAGTTCATGCTGCTAGCATTTACAGCTTTGCTT | - | KASP | KASP | Xie et al., 2020 |
|  |  | *Pm5e KASP-F2* | GAAGGTCGGAGTCAACGGATTGCTAGCATTTACAGCTTT |  |  |  |  |
|  |  | *Pm5e KASP-R* | GCAGTGGGAGCGTCATCAAA |  |  |  |  |
| *Pm6* | Coker747 | *CIT02g-18-F* | GGCCTTAGTGGTGATGCAGT | 300bp | PAGE | SSR | Wan et al., 2020 |
|  |  | *CIT02g-18-R* | GCGGCTTGTCGGTGTATAG |  |  |  |  |
| *Pm8* | Aurora | *sfr43-F* | TGGCTTCCAACAGCCCTAGC | 662bp | Agarose | - | Hurni et al., 2013 |
|  |  | *sfr43-R* | AGGCTTTTGCACCTTCTCTC |  |  |  |  |
| *Pm12* | CI14119 | *MBH1-F* | AGCTGTTTCCTTTCCAATGAGTAA | 330bp | PAGE | - | Zhu et al., 2023 |
|  |  | *MBH1-R* | GCTGTGAATCCATTATGCTGTTTCA |  |  |  |  |
| *Pm21* | Yangmai5 | *MBH2-F* | GCCATTATAGTCAAGAGTGCACTAGCTGT | 330bp | PAGE | EST | Bie et al., 2015 |
|  |  | *MBH2-R* | AGCTCCTCTCGTTCTCCAATGCT |  |  |  |  |
| *Pm24* | Chiyacao | *STS-Pm24-F* | TATGGTGTCATTTAAGGCTGAG | 171bp | PAGE | STS | Lu et al., 2020 |
|  |  | *STS-Pm24-R* | TTTCTCACATCCTCATCAAACC |  |  |  |  |
| *Pm35* | NC97BGTD7 | *CFD26-F* | TCAAGATCGTGCCAAATCAA | 268bp | PAGE | SSR | Miranda et al., 2007 |
|  |  | *CFD26-R* | ACTCCAAGCTGAGCACGTTT |  |  |  |  |
| *Pm37* | NC99BGTAG11 | *Pm37-82-F* | AGCAAAACCCTAAACCCTGG | - | PAGE | - | Su, unpublished |
|  |  | *Pm37-82-R* | ATATTCCAAGCGAACCATGC |  |  |  |  |
| *Pm41* | IW2 | *M171-F* | CGGAAGAAGATTTCTCCACATA | 1068bp | PAGE | SNP | Li et al., 2020 |
|  |  | *M171-R* | GCCGACCACTTGTCCACTT |  |  |  |  |
| *Pm42* | G-303-1M | *GWM148-F* | GTGAGGCAGCAAGAGAGAAA | 167bp | PAGE | SSR | Hua et al., 2009 |
|  |  | *GWM148-R* | CAAAGCTTGACTCAGACCAAA |  |  |  |  |
| *Pm45* | D57-6D | *CFD80-F* | ATAGGGGTTTTGAATCACTCC | 162bp | PAGE | SSR | Ma et al., 2011 |
|  |  | *CFD80-R* | TTGGATTTGCAGAGCCTTCT |  |  |  |  |
| *Pm47* | Hongyanglazi | *GWM46-F* | GCACGTGAATGGATTGGAC | 179bp | PAGE | SSR | Xiao et al., 2013 |
|  |  | *GWM46-R* | TGACCCAATAGTGGTGGTCA |  |  |  |  |
| *Pm52* | Liangxing99 | *ICSSL326-F* | AAGATGCACTTACCCAAAAAC | 149bp | PAGE | SSR | Wu et al., 2019 |
|  |  | *ICSSL326-R* | TGCTACATATAACTGCTGCTG |  |  |  |  |
| *Pm58* | TA1662 | *Xsts24035-F* | GCTGAGATAAACAACACCCTGACC | 118bp | PAGE | STS | Xue et al., 2022 |
|  |  | *Xsts24035-R* | TGGAGTGCGTGAAAATCCCC |  |  |  |  |
| *Pm59* | PI181356 | *Xmag1759-F* | GTACGAGTATGGCAACAGC | 205bp | PAGE | STS | Tan et al., 2018 |
|  |  | *Xmag1759-R* | CCAACTCTAGGGGCAGATG |  |  |  |  |
| *Pm60* | PI428309 | *M-Pm60-S1-F* | CTCACAGTTCCACACTGATAT | 831bp | Agarose | SSR | Zhao et al., 2020 |
|  |  | *M-Pm60-S1-R* | CTCCATCAATCTCAAGTTCTTCG |  |  |  |  |
|  |  | *M-Pm60-S2-F* | TGTATATTAATGGGTATAATAG | 756bp | Agarose | SSR |  |
|  |  | *M-Pm60-S2-R* | CTCCATCAATCTCAAGTTCTTCG |  |  |  |  |
| *Pm61* | XXSYH | *Xgwm160-F* | TTCAATTCAGTCTTGGCTTGG | 196bp | PAGE | SSR | Sun et al., 2018 |
|  |  | *Xgwm160-R* | TTCAATTCAGTCTTGGCTTGG |  |  |  |  |
| *Pm68* | TRI1796 | *Xdw12-F* | CAGAAACCTAGTCCCGACTGTTC | - | PAGE | SSR | He et al., 2020 |
|  |  | *Xdw12-R* | GATCGCTTCGATGGCCGAAGT |  |  |  |  |
|  |  | *Xdw15-F* | GCTAATTACTACTCTCTTCGTTCCGA | - | PAGE | SSR |  |
|  |  | *Xdw15-R* | GAATATGACCCAACAAATATCCGACA |  |  |  |  |
| *Pm69* | G305-3M | *uhw403-F* | GCTATCGCCATCTACCTATC | 549bp | Agarose | - | Li et al., 2022 |
|  |  | *uhw403-R* | ATAAACTCCCGAGATGCG |  |  |  |  |
| *Pm4* |  | *GH398* | CCTTCACACGGCAAATCTGAA | - | - | - | Sánchez-Martín et al., 2021 |
|  |  | *GH399* | GATGTGCACCCAACACTAACT |  |  |  |  |
|  |  | *GH400* | ATCAGAGTCTCTATCGCCCT |  |  |  |  |
|  |  | *GH401* | CACCCAACACTAACTGAAAGGAG |  |  |  |  |
|  |  | *GH407* | AGTAATAACTCTACGCAACATGAAG |  |  |  |  |
|  |  | *GH414* | TAGGTTGGAGAGATCACAACGA |  |  |  |  |
|  |  | *GH415* | CTGAGGTAGAGGAGGCAACTT |  |  |  |  |
|  |  | *GH377* | AGAGTGCAGAGACTTCAATCCA |  |  |  |  |
|  |  | *GH417* | TTCTTCGTACCCAGCAGGTC |  |  |  |  |

**Table S3 Detection of Powdery mildew (*Pm*) resistance genes detected with molecular markers**

| **Plant ID** | **Powdery mildew (*Pm*) resistance genes** | | | | | | | | | | | | | | | | | | | | |
| --- | --- | --- | --- | --- | --- | --- | --- | --- | --- | --- | --- | --- | --- | --- | --- | --- | --- | --- | --- | --- | --- |
|  | ***1*** | ***2*** | ***3*** | ***4*** | ***5e*** | ***6*** | ***8*** | ***12*** | ***21*** | ***24*** | ***35*** | ***37*** | ***41*** | ***42*** | ***52*** | ***58*** | ***59*** | ***60*** | ***61*** | ***68*** | ***69*** |
| CWI 4666 | - | - | - | - | - | - | - | - | - | - | - | - | - | - | - | - | - | - | - | - | - |
| CWI 4643 | - | - | - | - | - | - | - | - | - | - | - | - | - | - | - | - | - | - | - | - | - |
| CWI 48188 | - | - | - | - | - | - | - | - | - | - | - | - | - | - | - | - | - | - | - | - | - |
| CWI 80465 | - | - | - | - | - | - | - | - | - | - | - | - | - | - | - | - | - | - | - | - | - |
| CWI 80556 | - | - | - | - | - | - | - | - | - | - | - | - | - | - | - | - | - | - | - | - | - |
| CWI 83497 | - | - | - | - | - | - | - | - | - | - | - | - | - | - | - | - | - | - | - | - | - |
| CWI 96237 | - | - | - | - | - | - | - | - | - | - | - | - | - | - | - | - | - | - | - | - | - |
| CWI 96263 | - | - | - | - | - | - | - | - | - | - | - | - | - | - | - | - | - | - | - | - | - |
| CWI 96272 | - | - | - | - | - | - | - | - | - | - | - | - | - | - | - | - | - | - | - | - | - |
| CWI 96275 | - | - | - | - | - | - | - | - | - | - | - | - | - | - | - | - | - | - | - | - | - |
| CWI 96277 | - | - | - | - | - | - | - | - | - | - | - | - | - | - | - | - | - | - | - | - | - |
| CWI 96279 | - | - | - | - | - | - | - | - | - | - | - | - | - | - | - | - | - | - | - | - | - |
| CWI 96281 | - | - | - | - | - | - | - | - | - | - | - | - | - | - | - | - | - | - | - | - | - |
| CWI 96303 | - | - | - | - | - | - | - | - | - | - | - | - | - | - | - | - | - | - | - | - | - |
| CWI 96353 | - | - | - | - | - | - | - | - | - | - | - | - | - | - | - | - | - | - | - | - | - |
| CWI 52947 | - | - | - | - | - | - | - | - | - | - | - | - | - | - | - | - | - | - | - | - | - |
| CWI 19082 | - | - | - | - | - | - | - | - | - | - | - | - | - | - | - | - | - | - | - | - | - |
| CWI 19093 | - | - | - | - | - | - | - | - | - | - | - | - | - | - | - | - | - | - | - | - | - |
| CWI 19047 | - | - | - | - | - | - | - | - | - | - | - | - | - | - | - | - | - | - | - | - | - |
| CWI 19058 | - | - | - | - | - | - | - | - | - | - | - | - | - | - | - | - | - | - | - | - | - |
| CWI 53111 | - | - | - | - | - | - | - | - | - | - | - | - | - | - | - | - | - | - | - | - | - |
| CWI 16957 | - | - | - | - | - | - | - | - | - | - | - | - | - | - | - | - | - | - | - | - | - |
| CWI 16959 | - | - | - | - | - | - | - | - | - | - | - | - | - | - | - | - | - | - | - | - | - |
| CWI 17058 | - | - | - | - | - | - | - | - | - | - | - | - | - | - | - | - | - | - | - | - | - |
| CWI 17154 | - | - | - | - | - | - | - | - | - | - | - | - | - | - | - | - | - | - | - | - | - |
| CWI 18949 | - | - | - | - | - | - | - | - | - | - | - | - | - | - | - | - | - | - | - | - | - |
| CWI 19498 | - | - | - | - | - | - | - | - | - | - | - | - | - | - | - | - | - | - | - | - | - |
| CWI 19529 | - | - | - | - | - | - | - | - | - | - | - | - | - | - | - | - | - | - | - | - | - |
| CWI 19531 | - | - | - | - | - | - | - | - | - | - | - | - | - | - | - | - | - | - | - | - | - |
| CWI 19535 | - | - | - | - | - | - | - | - | - | - | - | - | - | - | - | - | - | - | - | - | - |
| CWI 19536 | - | - | - | - | - | - | - | - | - | - | - | - | - | - | - | - | - | - | - | - | - |
| CWI 2352 | - | - | - | - | - | - | - | - | - | - | - | - | - | - | - | - | - | - | - | - | - |
| CWI 38331 | - | - | - | - | - | - | - | - | - | - | - | - | - | - | - | - | - | - | - | - | - |
| CWI 5103 | - | - | - | - | - | - | - | - | - | - | - | - | - | - | - | - | - | - | - | - | - |
| CWI 6265 | - | - | - | - | - | - | - | - | - | - | - | - | - | - | - | - | - | - | - | - | - |
| CWI 83793 | - | - | - | - | - | - | - | - | - | - | - | - | - | - | - | - | - | - | - | - | - |
| CWI 83803 | - | - | - | - | - | - | - | - | - | - | - | - | - | - | - | - | - | - | - | - | - |
| CWI 17260 | - | - | - | - | - | - | - | - | - | - | - | - | - | - | - | - | - | - | - | - | - |
| CWI 18643 | - | - | - | - | - | - | - | - | - | - | - | - | - | - | - | - | - | - | - | - | - |
| CWI 18754 | - | - | - | - | - | - | - | - | - | - | - | - | - | - | - | - | - | - | - | - | - |
| CWI 18758 | - | - | - | - | - | - | - | - | - | - | - | - | - | - | - | - | - | - | - | - | - |
| CWI 4374 | - | - | - | - | - | - | - | - | - | - | - | - | - | - | - | - | - | - | - | - | - |
| CWI 4385 | - | - | - | - | - | - | - | - | - | - | - | - | - | - | - | - | - | - | - | - | - |
| CWI 4393 | - | - | - | - | - | - | - | - | - | - | - | - | - | - | - | - | - | - | - | - | - |
| CWI 4416 | - | - | - | - | - | - | - | - | - | - | - | - | - | - | - | - | - | - | - | - | - |
| CWI 5110 | - | - | - | - | - | - | - | - | - | - | - | - | - | - | - | - | - | - | - | - | - |
| CWI 93034 | - | - | - | - | - | - | - | - | - | - | - | - | - | - | - | - | - | - | - | - | - |
| CWI 93289 | - | - | - | - | - | - | - | - | - | - | - | - | - | - | - | - | - | - | - | - | - |
| CWI 94083 | - | - | - | - | - | - | - | - | - | - | - | - | - | - | - | - | - | - | - | - | - |
| CWI 94105 | - | - | - | - | - | - | - | - | - | - | - | - | - | - | - | - | - | - | - | - | - |
| CWI 94107 | - | - | - | - | - | - | - | - | - | - | - | - | - | - | - | - | - | - | - | - | - |
| CWI 94109 | - | - | - | - | - | - | - | - | - | - | - | - | - | - | - | - | - | - | - | - | - |
| CWI 94112 | - | - | - | - | - | - | - | - | - | - | - | - | - | - | - | - | - | - | - | - | - |
| CWI 94115 | - | - | - | - | - | - | - | - | - | - | - | - | - | - | - | - | - | - | - | - | - |
| CWI 94145 | - | - | - | - | - | - | - | - | - | - | - | - | - | - | - | - | - | - | - | - | - |
| CWI 94149 | - | - | - | - | - | - | - | - | - | - | - | - | - | - | - | - | - | - | - | - | - |
| CWI 94153 | - | - | - | - | - | - | - | - | - | - | - | - | - | - | - | - | - | - | - | - | - |
| CWI 94157 | - | - | - | - | - | - | - | - | - | - | - | - | - | - | - | - | - | - | - | - | - |
| CWI 94169 | - | - | - | - | - | - | - | - | - | - | - | - | - | - | - | - | - | - | - | - | - |
| CWI 94171 | - | - | - | - | - | - | - | - | - | - | - | - | - | - | - | - | - | - | - | - | - |
| CWI 96319 | - | - | - | - | - | - | - | - | - | - | - | - | - | - | - | - | - | - | - | - | - |
| CWI 17195 | - | - | - | - | - | - | - | - | - | - | - | - | - | - | - | - | - | - | - | - | - |
| CWI 80427 | - | - | - | - | - | + | - | - | - | - | - | - | - | - | - | - | - | - | - | - | - |
| CWI 80540 | - | - | - | - | - | + | - | - | - | - | - | - | - | - | - | - | - | - | - | - | - |
| CWI 17007 | - | - | - | - | - | + | - | - | - | - | - | - | - | - | - | - | - | - | - | - | - |
| CWI 17224 | - | - | - | - | - | + | - | - | - | - | - | - | - | - | - | - | - | - | - | - | - |
| CWI 17259 | - | - | - | - | - | + | - | - | - | - | - | - | - | - | - | - | - | - | - | - | - |
| CWI 17281 | - | - | - | - | - | + | - | - | - | - | - | - | - | - | - | - | - | - | - | - | - |
| CWI 18175 | - | - | - | - | - | + | - | - | - | - | - | - | - | - | - | - | - | - | - | - | - |
| CWI 18564 | - | - | - | - | - | + | - | - | - | - | - | - | - | - | - | - | - | - | - | - | - |
| CWI 18594 | - | - | - | - | - | + | - | - | - | - | - | - | - | - | - | - | - | - | - | - | - |
| CWI 14929 | - | - | - | - | - | - | - | - | - | - | - | - | - | - | - | - | - | - | - | - | - |
| CWI 17063 | - | - | - | - | - | - | - | - | - | - | - | - | - | - | - | - | - | - | - | - | - |
| CWI 17247 | - | - | - | - | - | - | - | - | - | - | - | - | - | - | - | - | - | - | - | - | - |
| CWI 17324 | - | - | - | - | - | - | - | - | - | - | - | - | - | - | - | - | - | - | - | - | - |
| CWI 17370 | - | - | - | - | - | - | - | - | - | - | - | - | - | - | - | + | - | - | - | - | - |
| CWI 17677 | - | - | - | - | - | - | - | - | - | - | - | - | - | - | - | - | - | - | - | - | - |
| CWI 17750 | - | - | - | - | - | - | - | - | - | - | - | - | - | - | - | - | - | - | - | - | - |
| CWI 17879 | - | - | - | - | - | - | - | - | - | - | - | - | - | - | - | - | - | - | - | - | - |
| CWI 17892 | - | - | - | - | - | - | - | - | - | - | - | - | - | - | - | - | - | - | - | - | - |
| CWI 17942 | - | - | - | - | - | - | - | - | - | - | - | - | - | - | - | - | - | - | - | - | - |
| CWI 17954 | - | - | - | - | - | - | - | - | - | - | - | - | - | - | - | - | - | - | - | - | - |
| CWI 17959 | - | - | - | - | - | - | - | - | - | - | - | - | - | - | - | - | - | - | - | - | - |
| CWI 17964 | - | - | - | - | - | - | - | - | - | - | - | - | - | - | - | - | - | - | - | - | - |
| CWI 18042 | - | - | - | - | - | - | - | - | - | - | - | - | - | - | - | - | - | - | - | - | - |
| CWI 18043 | - | - | - | - | - | - | - | - | - | - | - | - | - | - | - | - | - | - | - | - | - |
| CWI 18065 | - | - | - | - | - | - | - | - | - | - | - | - | - | - | - | - | - | - | - | - | - |
| CWI 18067 | - | - | - | - | - | - | - | - | - | - | - | - | - | - | - | - | - | - | - | - | - |
| CWI 18080 | - | - | - | - | - | - | - | - | - | - | - | - | - | - | - | - | - | - | - | - | - |
| CWI 18090 | - | - | - | - | - | - | - | - | - | - | - | - | - | - | - | - | - | - | - | - | - |
| CWI 18143 | - | - | - | - | - | - | - | - | - | - | - | - | - | - | - | - | - | - | - | - | - |
| CWI 18165 | - | - | - | - | - | - | - | - | - | - | - | - | - | - | - | - | - | - | - | - | - |
| CWI 18166 | - | - | - | - | - | - | - | - | - | - | - | - | - | - | - | - | - | - | - | - | - |
| CWI 18353 | - | - | - | - | - | - | - | - | - | - | - | - | - | - | - | - | - | - | - | - | - |
| CWI 18371 | - | - | - | - | - | - | - | - | - | - | - | - | - | - | - | - | - | - | - | - | - |
| CWI 18434 | - | - | - | - | - | - | - | - | - | - | - | - | - | - | - | + | - | - | - | + | - |
| CWI 18439 | - | - | - | - | - | - | - | - | - | - | - | - | - | - | - | + | - | - | - | + | - |
| CWI 18453 | - | - | - | - | - | - | - | - | - | - | - | - | - | - | - | - | - | - | - | - | - |
| CWI 18468 | - | + | - | - | - | - | - | - | - | - | - | - | - | - | - | - | - | - | - | - | - |
| CWI 18475 | - | - | - | - | - | - | - | - | - | - | - | - | - | - | - | + | - | - | - | - | - |
| CWI 18476 | - | - | - | - | - | - | - | - | - | - | - | - | - | - | - | + | - | - | - | - | - |
| CWI 18477 | - | - | - | + | - | - | - | - | - | - | - | - | - | - | - | - | - | - | - | - | - |
| CWI 19131 | - | - | - | - | - | - | - | - | - | - | - | - | - | - | - | - | - | - | - | - | - |
| CWI 44151 | - | - | - | - | - | - | - | - | - | - | - | - | - | - | - | - | - | - | - | - | - |
| CWI 44154 | - | - | - | - | - | - | - | - | - | - | - | - | - | - | - | + | - | - | - | - | - |
| CWI 44183 | - | - | - | - | - | - | - | - | - | - | - | - | - | - | - | - | - | - | - | - | - |
| CWI 44215 | - | - | - | - | - | - | - | - | - | - | - | - | - | - | - | + | - | - | - | - | - |
| CWI 44218 | - | - | - | - | - | - | - | - | - | - | - | - | - | - | - | + | - | - | - | - | - |
| CWI 44355 | - | - | - | - | - | - | - | - | - | - | - | - | - | - | - | - | - | - | - | - | - |
| CWI 44394 | - | - | - | - | - | - | - | - | - | - | - | - | - | - | - | - | - | - | - | - | - |
| CWI 44396 | - | - | - | - | - | - | - | - | - | - | - | - | - | - | - | - | - | - | - | - | - |
| CWI 44398 | - | - | - | - | - | - | - | - | - | - | - | - | - | - | - | - | - | - | - | - | - |
| CWI 44403 | - | - | - | - | - | - | - | - | - | - | - | - | - | - | - | - | - | - | - | - | - |
| CWI 44409 | - | - | - | - | - | - | - | - | - | - | - | - | - | - | - | + | - | - | - | - | - |
| CWI 44410 | - | - | - | - | - | - | - | - | - | - | - | - | - | - | - | - | - | - | - | - | - |
| CWI 78968 | - | - | - | - | - | - | - | - | - | - | - | - | - | - | - | - | - | - | - | - | - |
| CWI 78972 | - | - | - | - | - | - | - | - | - | - | - | - | - | - | - | - | - | - | - | - | - |
| CWI 78976 | - | - | - | + | - | - | - | - | - | - | - | - | - | - | - | - | - | - | - | - | - |
| CWI 78977 | - | - | - | + | - | - | - | - | - | - | - | - | - | - | - | - | - | - | - | - | - |
| CWI 78979 | - | - | - | - | - | - | - | - | - | - | - | - | - | - | - | - | - | - | - | - | - |
| CWI 78980 | - | - | - | - | - | - | - | - | - | - | - | - | - | - | - | - | - | - | - | - | - |
| CWI 78981 | - | - | - | - | - | - | - | - | - | - | - | - | - | - | - | - | - | - | - | - | - |
| CWI 78982 | - | - | - | - | - | - | - | - | - | - | - | - | - | - | - | - | - | - | - | - | - |
| CWI 78983 | - | - | - | - | - | - | - | - | - | - | - | - | - | - | - | - | - | - | - | - | - |
| CWI 78984 | - | - | - | - | - | - | - | - | - | - | - | - | - | - | - | - | - | - | - | - | - |
| CWI 78985 | - | - | - | - | - | - | - | - | - | - | - | - | - | - | - | - | - | - | - | - | - |
| CWI 78986 | - | - | - | - | - | - | - | - | - | - | - | - | - | - | - | - | - | - | - | - | - |
| CWI 78989 | - | - | - | - | - | - | - | - | - | - | - | - | - | - | - | - | - | - | - | - | - |
| CWI 78990 | - | - | - | - | - | - | - | - | - | - | - | - | - | - | - | - | - | - | - | - | - |
| CWI 78991 | - | - | - | - | - | - | - | - | - | - | - | - | - | - | - | - | - | - | - | - | - |
| CWI 80462 | - | - | - | - | - | - | - | - | - | - | - | - | - | - | - | + | - | - | - | - | - |
| CWI 80503 | - | - | - | - | - | - | - | - | - | - | - | - | - | - | - | + | - | - | - | - | - |
| CWI 80504 | - | - | - | - | - | - | - | - | - | - | - | - | - | - | - | - | - | - | - | - | - |
| CWI 80531 | - | - | - | + | - | - | - | - | - | - | - | - | - | - | - | - | - | - | - | - | - |
| CWI 87084 | - | - | - | - | - | - | - | - | - | - | - | - | - | - | - | - | - | - | - | - | - |
| CWI 92915 | - | - | - | - | - | - | - | - | - | - | - | - | - | - | - | + | - | - | - | - | - |
| CWI 93151 | - | - | - | - | - | - | - | - | - | - | - | - | - | - | - | - | - | - | - | - | - |

Note: +: gene present; -: gene absent.
